# Enhanced Strength of a Mechanical Alloyed NbMoTaWVTi Refractory High Entropy Alloy

**DOI:** 10.3390/ma11050669

**Published:** 2018-04-25

**Authors:** Yan Long, Kai Su, Jinfu Zhang, Xiaobiao Liang, Haiyan Peng, Xiaozhen Li

**Affiliations:** 1 Guangdong Key Laboratory for Processing and Forming of Advanced Metallic Materials, School of Mechanical and Automotive Engineering, South China University of Technology, Guangzhou 510640, China; 201620101447@mail.scut.edu.cn (K.S.); changchinfu@163.com (J.Z.); mehypeng@mail.scut.edu.cn (H.P.); 2 National Metallic Materials Near-net-shape Forming Engineering Research Center, Guangzhou 510640, China; 17876565708@163.com (X.-b.L.); Jane1517@163.com (X.-z.L.)

**Keywords:** refractory high entropy alloy, microstructure, mechanical properties

## Abstract

A NbMoTaWVTi refractory high entropy alloy (HEA) has been successfully synthesized by mechanical alloying (MA) and spark plasma sintering (SPS). The microstructure and mechanical properties of this alloy are investigated. It is observed that only two types of body-centered cubic (BCC) solid solutions are formed in the powders after ball milling for 40 h. However, a new face-centered cubic (FCC) precipitated phase is observed in the BCC matrix of bulk material consolidated by SPS. The FCC precipitated phase is identified as TiO, due to the introduction of O during the preparing process of HEA. The compressive yield strength, fracture strength, and total fracture strain of the consolidated bulk HEA are 2709 MPa, 3115 MPa, and 11.4%, respectively. The excellent mechanical properties can be attributed to solid solution strengthening and grain boundary strengthening of the fine-grained BCC matrix, as well as the precipitation strengthening owing to the formation of TiO particles.

## 1. Introduction

According to the traditional alloy design theory, ordered phases or intermetallic compounds will form as the number of alloying elements increases. However, Yeh et al. observes that simple solid solution phases, instead of intermetallic compounds, are formed in an equiatomic quinary alloy. Subsequently, they propose a new alloy system, termed high entropy alloys (HEAs), which contain at least five principal elements, each having the atomic percentage between 5% and 35% [1,2]. Since the development of HEAs, a substantial number of studies on HEA have been performed. Some of these studies are focused on alloys comprised of elements with low melting points, such as AlCoCrCuFeNi system alloys [3,4], commonly exhibiting high ductility and low strength. On the other hand, refractory HEAs have exhibited advantages of high yield strength at room and elevated temperature. For example, Nb25Mo25Ta25W25 and V20Nb20Mo20Ta20 refractory HEAs produced by vacuum arc-melting exhibit compressive yield strengths of 1058 MPa and 1246 MPa, respectively, with plasticities of less than 2% at room temperature [5]. Han et al. have successfully synthesized TiNbMoTaW and TiVNbMoTaW refractory HEAs. It is found that the addition of Ti with the largest atomic size significantly improves the mechanical properties of HEAs at room and elevated temperature [6,7].

Bulk HEAs are usually prepared by vacuum arc-melting technology, with the disadvantages of coarse grains and dendritic segregation [3,5]. By comparison, it is a promising method to use spark plasma sintering (SPS) to consolidate mechanically alloyed (MA) HEA powders, which can produce high-performance bulk HEAs with homogeneous structure and finer grains [8]. However, it is inevitable that interstitial elements, such as O, N, etc. will be introduced during the preparing process, which will lead to the formation of precipitated phases [4,8,9]. In fact, several recent studies have revealed that in many cases, dual-phase microstructures can be observed in some HEAs [10,11] and the second phase significantly enhances the strength of HEAs by precipitation strengthening [12,13,14,15,16,17]. Therefore, the precipitated phase, caused by introduction of interstitial elements, may benefit the mechanical properties of HEAs in some cases. In the present work, a multi-phase NbMoTaWVTi refractory HEA was fabricated by MA and SPS, and the microstructure evolution and mechanical properties of the sintered HEA are subsequently investigated.

## 2. Experimental Methods

Nb, Mo, Ta, W, V, Ti elemental powders with high purity (≥99.9%) are mixed in equiatomic ratio, and subsequently milled in a high energy planetary ball mill. Stainless steel balls and vial are used for the ball milling of mixed powders at the ball-to-powder weight ratio of 13:1, and the vial is filled with high purity argon to protect powders from excess oxidation. At milling times of 5, 10, 20, 30, and 40 h, a portion of ball milled powders are taken out for analysis, and the rest of milled powders are removed after 40 h. The powders milled for 40 h are loaded into a graphite die. Graphite foils are placed between the powders and the die walls, to prevent mutual fusing. These powders are subsequently heated by a spark plasma sintering machine (SPS-825, Sumitomo Coal Mining Co. Ltd., Tokyo, Japan) with a heating rate of 100 °C/min, and preserved at 1400 °C for 10 min under a pressure of 30 MPa. After high-temperature sintering, the heated specimen is cooled to 500 °C with a cooling rate of 300 °C/min, and then slowly cooled down to ambient temperature. Finally, a cylinder specimen with the diameter of 20 mm and height of 10 mm is obtained.

Archimedes’ method is used for density measurements. The powders and sintered sample are tested by X-ray diffraction (XRD, Bruker D8 Advance, Billerica, MA, USA) with a Cu Kα radiation. The oxygen content in milled powders is measured by ICP-AES ULTMA2, SPECTRO Analytical Instruments Inc, Mahwah, NJ, USA. The thermal analysis is performed in a differential scanning calorimeter (DSC, Netzsch Sta 449C, Bavarian, Germany) at a heating rate of 10 K/min under flowing high purity argon atmosphere. Microstructures of sintered samples are observed by a scanning electron microscope (SEM, FEI Nano 430, Hillsboro, OR, USA). Thin foil specimens are prepared by mechanical thinning, followed by ion milling, and are observed under a transmission electron microscopy (TEM, FEI G2 F20, Hillsboro, OR, USA). Cylindrical samples of Φ3 mm × 6 mm are prepared for compressive tests at room temperature. The compressive test is accomplished by AG-100kNX universal testing machine at a strain rate of 1 × 10^−3^ s^−1^.

## 3. Results and Discussion

### 3.1. Microstructure and Phase Transition

**Figure 1:**
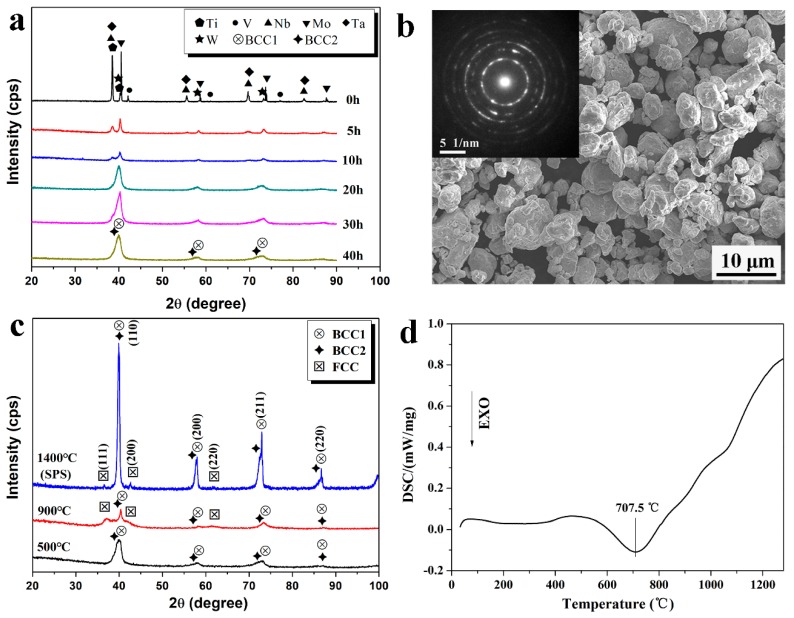
(**a**) XRD patterns of NbMoTaWVTi powders with different milling times; (**b**) SEM image and corresponding SAED pattern of the high entropy alloy (HEA) powders after 40 h of milling; (**c**) XRD patterns of ball milled powders heat-treated at 500 °C, 900 °C for 1 h, and bulk HEA after spark plasma sintering (SPS) at 1400 °C; (**d**) DSC curve of the HEA powders after 40 h of milling.

Figure 1a shows the XRD patterns of the equiatomic NbMoTaWVTi high entropy alloy powders with different milling times. Diffraction peaks of all the pure elements can be observed in the initial blend. At the beginning of ball milling, the peak intensity of Ti, Nb, Ta, and V decrease more rapidly than W and Mo. Subsequently, two overlapping peaks near the peaks of W and Mo, characterized as body-centered cubic (BCC) solutions, are observed in the XRD pattern after 20 h. It can be hypothesized that the solid solution sequence of the elements is related to the melting point, with low-melting-point elements being dissolved more quickly than the elements with higher melting points, which has been similarly reported in the previous work [18]. In order to obtain alloy powders with homogeneous composition, the milling time is prolonged to 40 h. The equiatomic NbMoTaWVTi HEA powders after 40 h of milling had an average particle size of less than 10 μm, as shown in Figure 1b, and the SAED pattern of them is shown in the inset of Figure 1b. It confirms that the powders after 40 h of milling have only BCC structures, which is in accordance with the XRD pattern.

However, a new FCC phase is observed in the bulk HEA consolidated by SPS, according to the XRD pattern shown in Figure 1c. The BCC1 phases and the FCC phase reveal lattice parameters of approximately 3.172 Å and 4.235 Å, respectively. In order to confirm the phase transition during the SPS process, DSC analysis and heat treatment of milled powders are carried out. Figure 1d presents the DSC curve that an exothermic peak is obviously observed at 707.5 °C. It can be derived that phase transition has occurred during the heating process of the milled powders. Figure 1c also shows the XRD patterns of powders after heat treatment at 500 °C and 900 °C for 1 h, respectively. The XRD pattern of powders after heating at 500 °C is nearly the same as the original powders, while new diffraction peaks, characterized as face-centered cubic (FCC) structures, are observed both in powders heated at 900 °C and consolidated bulk HEA. Thus, it is reasonable to conclude that a new FCC phase precipitates from original BCC phases during the SPS process.

**Figure 2:**
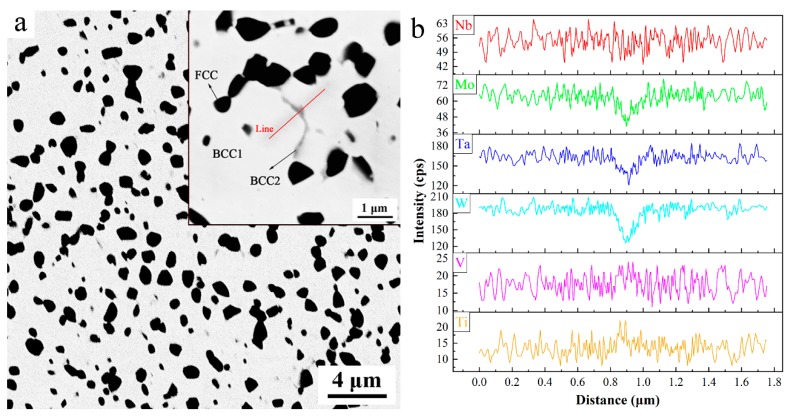
(**a**) SEM back scatter electron images of the bulk NbMoTaWVTi HEA at different magnifications and (**b**) Elemental composition gradients along the red drawn line.

Figure 2a illustrates the SEM backscattered images of bulk NbMoTaWVTi alloy at different magnifications and elemental composition gradients along the drawn red line. It is clear that the sintered bulk alloy consists of a white matrix phase (BCC1), a black precipitated phase (FCC), and a few gray intergranular phases (BCC2). The average size and volume fraction of FCC particles are measured to be 0.64 μm and 16.1%, respectively. The thickness of BCC2 phase is measured to be about 60~250 nm, and the volume fraction of BCC2 is estimated to be about 3%. The chemical compositions of the matrix phase and the precipitated phase are determined by the EDS quantitative analysis. The results shown in Table 1 indicate that the BCC1 phase of the NbMoTaWVTi HEA is depleted of Ti, and has nearly equivalent amounts of all the other elements, while the precipitated phase basically contains Ti and O. However, the BCC2 phase is too small to be determined by the EDS quantitative analysis. Therefore, line scanning across grain boundaries is carried out to identify the chemical distribution of BCC2 phase. The results shown in Figure 2b indicate that the chemical composition of BCC2 phase is close to BCC1, except that it is slightly depleted of W, Mo, and Ta. It can be speculated that the formation of BCC2 results from the slightly inhomogeneous distribution of alloying elements at the grain boundaries of sintered HEA.

**Figure 3:**
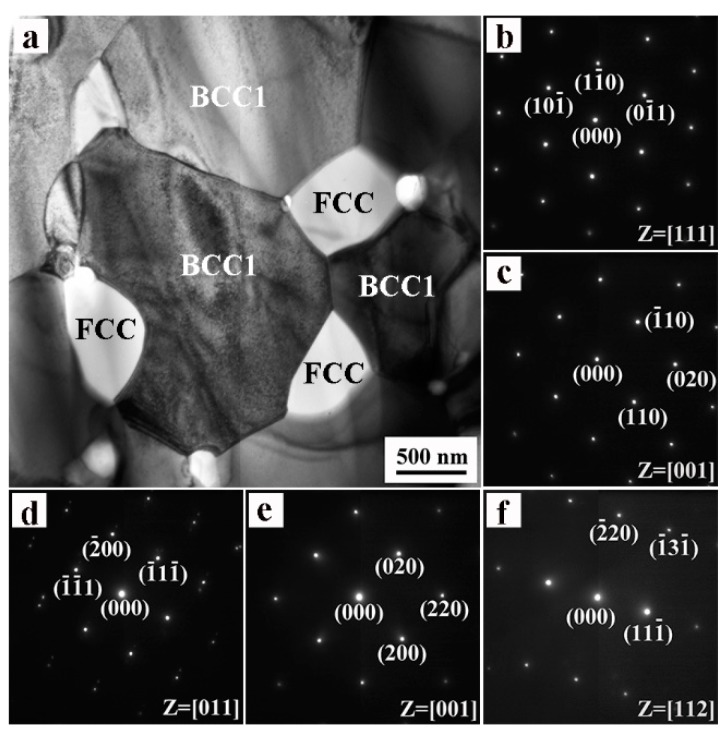
TEM images of the bulk NbMoTaWVTi HEA: (**a**) Bright-field image; (**b**,**c**) SAED patterns of BCC1 phase along [111] and [001] zone axis, respectively; (**d**–**f**) SAED patterns of FCC phase along [011], [001] and [112] zone axis, respectively.

Figure 3 shows a bright-field TEM micrograph and selected area electron diffraction (SAED) patterns of the bulk NbMoTaWVTi alloy after SPS. It can be observed that the bulk HEA basically consists of a BCC1 matrix phase with an average grain size of 1.35 μm and an FCC precipitated phase. The precipitated phase is basically distributed at the grain boundaries of the BCC matrix. Chemical composition of phases in Figure 3a is measured by EDS/TEM. It is found that the FCC phase primarily contained Ti and O, with a Ti to O atomic ratio of approximately 1:1. Considering the XRD diffraction peaks of FCC phase in Figure 1c are very close to that of TiO, the FCC precipitated phase can be confirmed to be TiO. It is of particular interest to note that TiO has an FCC structure at elevated temperature and a monoclinic crystal structure at ambient temperature [19]. The formation of TiO phase with FCC structure in the sintered sample indicated that the TiO phase remains in its original crystal structure at elevated temperature, due to the rapid cooling speed after SPS. There is a high probability that oxygen may be brought into the HEA during the MA process, as previously reported [8]. The mass fraction of oxygen in the powders after 40 h of milling is measured to be 0.86%. Yeh et al. [1] have pointed out that the high-entropy theory does not apply to materials with very large heats of formation, such as oxides, carbides, and nitrides. It is apparent that Ti has a much higher oxygen affinity than other alloying elements, therefore leading to the formation of TiO phase.

### 3.2. Mechanical Properties

**Figure 4:**
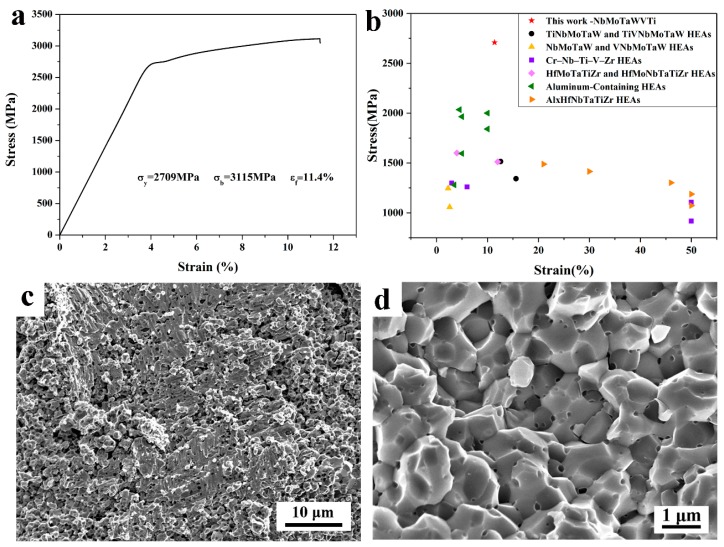
(**a**) Compressive engineering stress–strain curve of the bulk NbMoTaWVTi HEA; (**b**) Compressive yield strength versus fracture strain of the bulk HEA in the present work and available data in the literature; (**c**) Fracture morphologies of bulk HEA at low magnification; (**d**) High-magnified image of intergranular fracture area.

The density of bulk NbMoTaWVTi HEA consolidated by SPS was 10.6 g/cm^3^, and the theoretical density of a disordered solid solution, $\rho_{th}$, is calculated to be 10.96 g/cm^3^ by

(1) $$\rho_{th}=\frac{\sum C_{i}A_{i}}{\sum \frac{C_{i}A_{i}}{\rho_{i}}}$$

where $A_{i}$ and $\rho_{i}$ are the atomic weight and density of element *i*, respectively. The relative density is calculated to be 96.75%. Furthermore, the microhardness of bulk NbMoTaWVTi HEA is measured to be 786 HV, which is larger than 510 HV of the as-cast NbMoTaWVTi HEA [6]. Figure 4a shows the compressive engineering stress–strain curve of the sintered HEA specimen. The alloy exhibits compressive yield strength of 2709 MPa, fracture strength of 3115 MPa, and total fracture strain of 11.4% at ambient temperature.

The mechanical properties are compared with other reported refractory HEAs [5,6,20,21,22,23], as shown in Figure 4b. It is apparent that the sintered NbMoTaWVTi HEA possesses the highest yield strength and fair fracture strain. Notably, the compressive yield strength and fracture strength of the NbMoTaWVTi HEA are 78.8% and 45.9%, respectively, higher than those of the corresponding as-cast HEA, while the ductility decreases about 20% [12]. Compared with the coarse dendrite grains with the average grain size of tens of microns in the as-cast HEA, the equiaxed BCC matrix grains with the average grain size of 1.35 μm in the present work is much finer, therefore leading to a strong grain boundary strengthening effect. On the other hand, the precipitated fine TiO particles are likely to impede the dislocation motion in the BCC matrix, thus increasing strength and slightly decreasing the ductility of the alloy, simultaneously. It should be noted that the solid solution strengthening caused by atomic size difference of alloying elements in the HEA also contributes to the excellent mechanical properties of the NbMoTaWVTi HEA.

Figure 4c,d shows fracture morphologies of NbMoTaWVTi HEA at different magnifications. There are two types of morphologies in the fracture surface: flat and smeared area, as a result of shear tearing, and intergranular fracture area, with some fine particles dispersed on the grain boundaries, indicating a combination of plastic and brittle fracture mechanisms.

## 4. Conclusions

Equiatomic NbMoTaWVTi refractory HEA has been successfully synthesized by MA and SPS. It exhibits excellent mechanical properties with compressive yield strength of 2709 MPa, fracture strength of 3115 MPa and fracture strain of 11.4%. The sintered alloy consists of a BCC matrix phase, a minor BCC phase, and an FCC precipitated phase, identified as TiO. The average grain size of BCC matrix phase and TiO are measured to be 1.35 μm and 0.46 μm, respectively. Compared with the as-cast NbMoTaWVTi alloy, the compressive yield strength and fracture strength of the alloy in this work are extraordinarily elevated, while the ductility is slightly reduced. Thus, mechanical alloying and spark plasma sintering is a promising way to further improve mechanical properties of the refractory HEA by means of grain refinement and precipitation strengthening.

## Figures and Tables

**Table 1:** Chemical composition (in atom %) analysis results of white matrix phase (BCC1) and black precipitated phase (FCC) in Figure 2 by EDS/SEM.

Alloy and Phases	Ti	V	Nb	Mo	Ta	W	O
Nominal	16.67	16.67	16.67	16.67	16.67	16.67	-
BCC1	8.09	18.91	17.89	18.76	18.38	17.96	-
FCC	41.68	4.92	3.02	2.92	4.19	3.94	39.3
